# Transient anterior chamber intraocular lens opacification by triamcinolone acetonide following intravitreal injection

**DOI:** 10.3205/oc000248

**Published:** 2024-12-03

**Authors:** Kshitiz Kumar, Deepak Agarwal, Aditya Bajaj, Subrata Saha

**Affiliations:** 1 Department of Vitreo-Retina, Disha Eye Hospital, Kolkata, India; 2 Department of Ophthalmology, Disha Eye Hospital, Siliguri, India

**Keywords:** ACIOL, triamcinolone acetonide, pseudohypopyon, CME

## Abstract

**Background:**

Pseudophakic cystoid macular edema (CME) following primary anterior-chamber intraocular lens (ACIOL) implantations is commonly seen. Intravitreal triamcinolone acetonide (IVTA) injections have shown significant improvement in visual acuity and retinal thickness in refractory pseudophakic CME. Pseudohypopyon following IVTA injection is a known entity.

**Objective:**

To report a rare complication of transient acute vision loss due to ACIOL coating with triamcinolone acetonide particles following intravitreal injection.

**Methods:**

Case study

**Results:**

A patient developed pseudophakic cystoid macular edema two months post complicated cataract operation with ACIOL implantation. Despite topical steroid treatment, CME persisted and the patient was administered intravitreal triamcinolone acetonide injection. Acute vision loss due to dense coating of ACIOL with triamcinolone particles was noted on the next day. Conservative management led to spontaneous clearing of intraocular lens (IOL) and resolution of CME in 4 weeks.

**Conclusions:**

Opacification of ACIOL following IVTA injection is a rare complication which can be seen in eyes with compromised zonular/capsular bag integrity.

## Introduction

Cystoid macular edema (CME) post-cataract surgery, also called Irvine-Gass syndrome, is the most common cause of decreased vision in the early post-operative period. The incidence of clinical pseudophakic CME, defined by symptomatic vision loss, is reported at 1.17%–4.04% and can reach as high as 10.9% on optical coherence tomography (OCT) based diagnosis [1], [2], whereas in eyes with primary anterior-chamber intraocular lens (ACIOL) implantations, CME incidence is between 5.7%–22.2% [3]. Intravitreal triamcinolone acetonide injections have shown significant improvement in visual acuity and retinal thickness in refractory pseudophakic CME [4]. Pseudohypopyon/noninfectious endophthalmitis represents dispersion of triamcinolone acetonide crystals in the anterior chamber (AC) and vitreous cavity or an acute inflammatory reaction to a component in the drug formulation with an incidence ranging 0.2%–1.6%, and may show spontaneous resolution [5]. Massive AC transit of triamcinolone without serious adverse effect on corneal endothelium has been reported only once [6]. Opaque coating of triamcinolone acetonide on intraocular lens and iris leading to drop in vision was seen following injection of triamcinolone into AC [7].

This case report describes a rare complication of transient acute vision loss due to ACIOL coating with triamcinolone acetonide particles following intravitreal injection. 

## Case description

**Figure 1:**
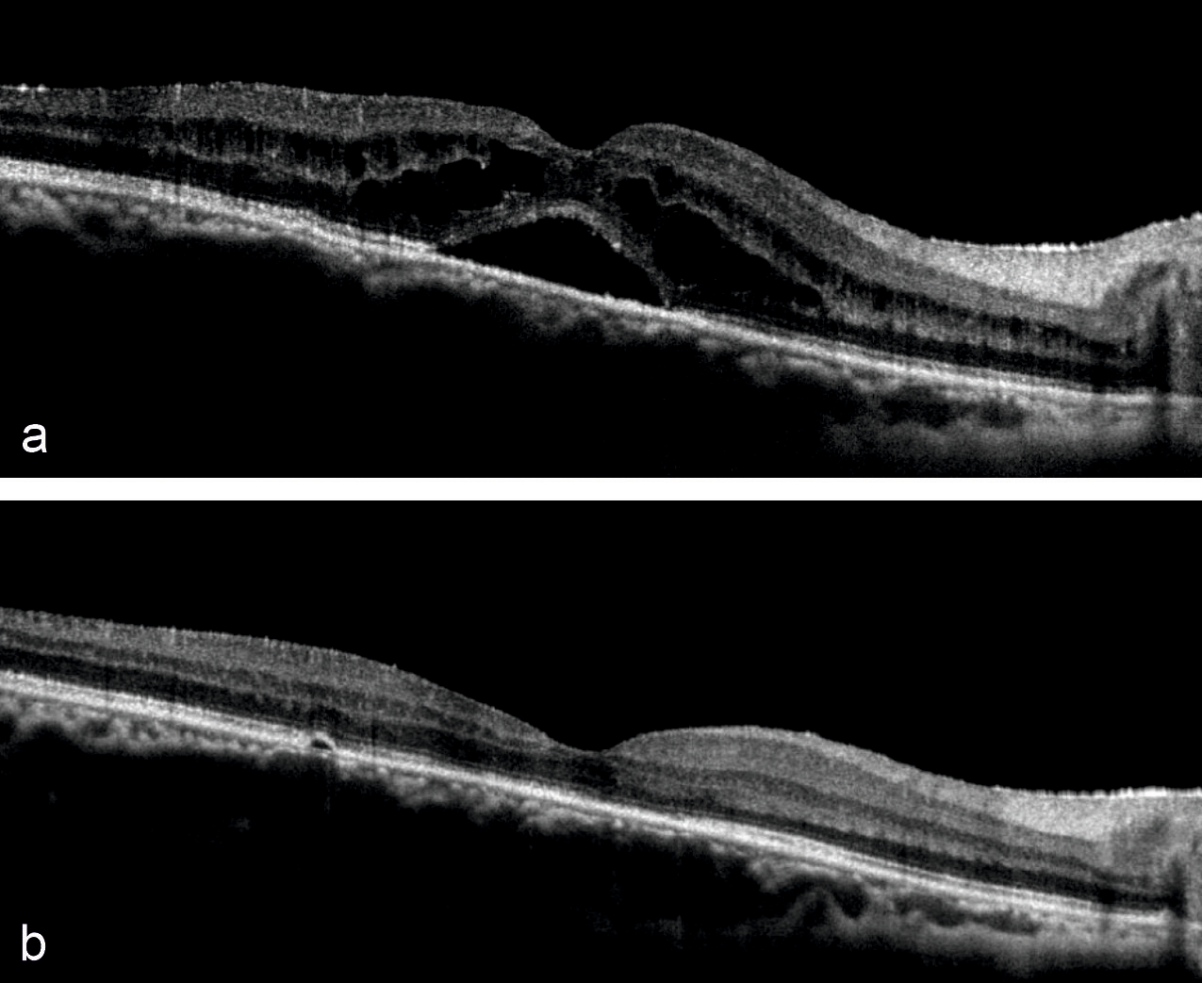
SD-OCT images of right eye horizontal section showing (a) CME with NSD before IVTA injection and (b) resolved macular edema post-injection at 3 weeks

**Figure 2:**
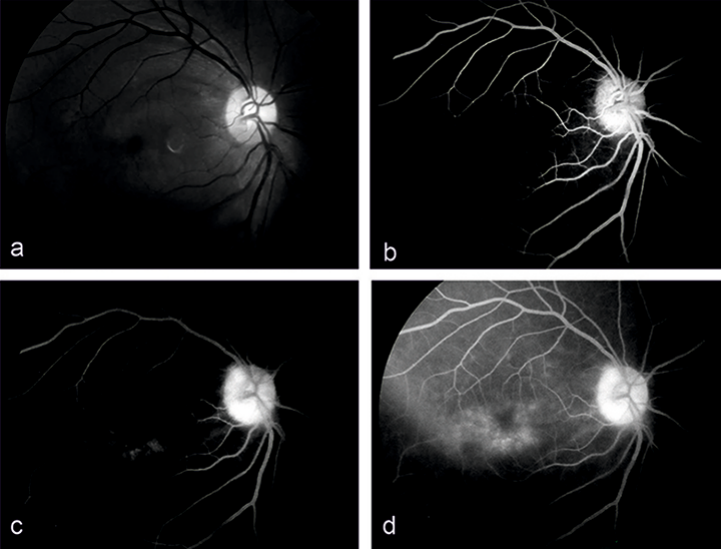
Red free fundus image of right eye (a) showing dull foveal reflex. Fundus fluorescein angiography images showing (b) early phase hyperfluorescence in macula, (c) diffuse hyperfluorescence in late mid-phase and (d) petaloid pattern macular leakage in late phase

**Figure 3:**
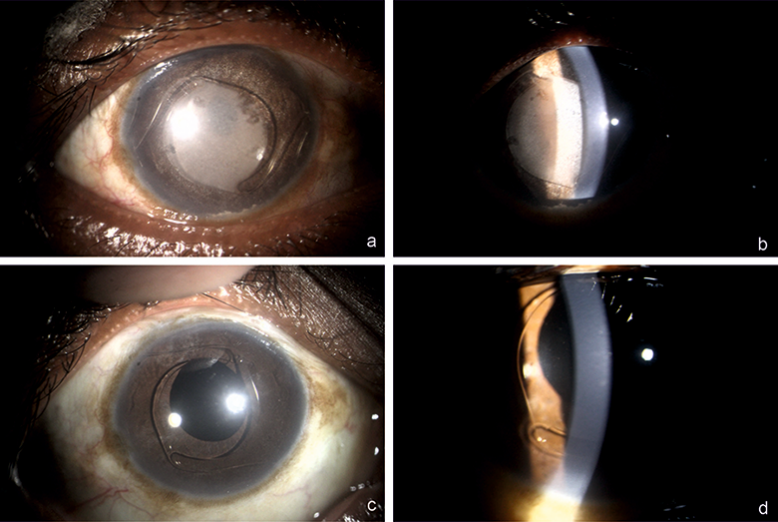
Slit-lamp photography of right eye. Post-op day 1 images (a & b) showing under diffuse and optical section illumination-suspended particles, inferior AC precipitates and dense coating of ACIOL with IVTA; 3 weeks images (c & d) showing quiet AC and clear ACIOL devoid of any particulate matter

**Figure 4:**
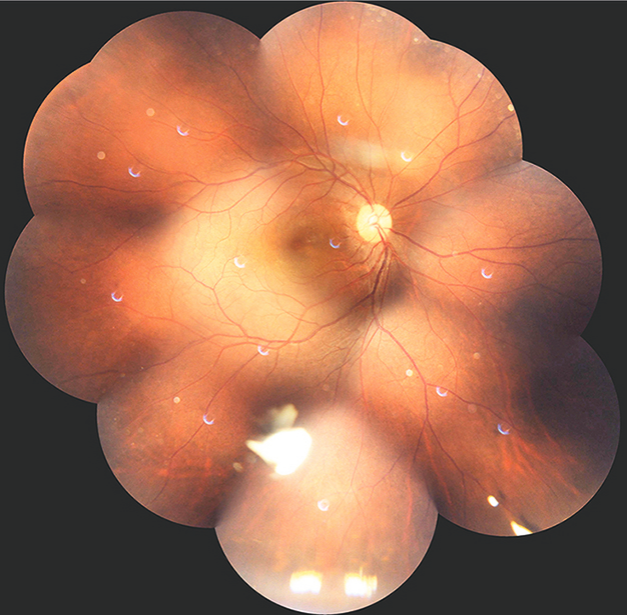
Fundus montage imaging showing suspended triamcinolone acetonide matter in inferior vitreous and normal retinal appearance

A 37-year-old male presented 2 months post-cataract surgery with right eye (RE) diminution of vision and best corrected visual activity (BCVA) 20/60. Slit-lamp examination of the anterior chamber showed ACIOL. Fundus evaluation revealed macular edema in RE which was confirmed on spectral-domain optical coherence tomography as CME. CME persisted with neurosensory detachment (547 microns) despite on topical steroid treatment at 6 weeks (Figure 1a (Fig. 1)). Fluorescein angiography confirmed petaloid macular leakage (Figure 2 (Fig. 2)). Intravitreal triamcinolone acetonide (IVTA, 0.05 ml) was injected in RE. Immediate post-op day 1 patient came back with severe drop in vision to hand movement close to face (HMCF). Slit-lamp photography showed clear cornea, particulate AC flare, inferior AC precipitates with dense coating of ACIOL with IVTA particles and no view of fundus (Figure 3a and b (Fig. 3)). Intraocular pressure (IOP) was 14 mmHG in RE. Patient was counselled about the complication and treated conservatively with topical eye drop timolol under close observation. At 1 week, intraocular lens (IOL) surface started clearing, by 3 weeks ACIOL had become devoid of any deposits (Figure 3c and d (Fig. 3)) and CME had resolved with BCVA of 20/40 (252 microns) (Figure 1b (Fig. 1)). Montage fundus image showed suspended IVTA particles inferiorly with normal optic disc and retina (Figure 4 (Fig. 4)).

## Discussion

Adverse effects commonly associated with IVTA procedures are IOP increase, cataract formation or progression, and pseudohypopyon/non-infectious endophthalmitis [8]. Characteristically the latter shows absence of pain, eyelid edema, no increased conjunctival injection and presence of corneal edema in the immediate post-op period [5]. This case mimicked pseudohypopyon but the cornea was clear indicating a non-inflammatory etiology. Prior to this report, there has been only a single case describing massive transit of triamcinolone acetonide (TA) particles into anterior chamber following intravitreal injection but in a phakic eye. The case required irrigation of AC and took 3 months for resolution [6], whereas this case had ACIOL with posterior capsular defect and the amount of TA was not much to warrant surgical removal, and faster resolution was seen on observation only. This case report is the first known incidence of ACIOL coating by triamcinolone acetonide resulting in transient loss of vision following intravitreal injection for pseudophakic CME. Chen et al. reported dense coating of posterior chamber intraocular lens (PCIOL) and iris stroma following intracameral injection of TA with resulting regression of neovascularization of the iris (NVI) at 3 months [7]. Again this case reiterates the fact that anterior migration and deposition of TA in eyes without capsular support/weak zonules does not induce inflammation and adversely affects cornea or iris. Anterior chamber dislocation or migration of dexamethasone implant (DEX-I, Ozurdex) through the pupil in aphakic eyes, or through an iridectomy, and around an intraocular lens are being reported frequently with serious adverse effect on corneal endothelium and corneal edema or decompensation necessitating its urgent surgical removal [9]. Röck et al. identified eyes with aphakia or vitrectomized pseudophakic eyes with reduced zonular/capsular bag complex integrity having high incidence of DEX-I migration into anterior chamber [9]. Apart from weak zonules and posterior capsular defect, history of vitrectomy increases the chances of this implant migration manifold. The vitreous cavity in such eyes is filled with aqueous fluid and therefore the implant has the potential to migrate forth and back with minimal resistance and changing postures. In addition, intraocular aqueous currents may carry the relatively buoyant and mobile implant from the posterior segment to the anterior segment in a vitrectomized eye if the implant is not anchored by residual vitreous [10]. Based on the above mechanisms, in this case as well, partially vitrectomized state was unable to hold some amount of injected TA particles in the posterior chamber and existing aqueous currents facilitated the migration of loose TA into anterior chamber across the pupil. In such high-risk cases where migration of DEX-I or IVTA into anterior chamber is a likely possibility, subconjunctival TA injections offer a safe alternative for treating macular edema [11].

Thus to conclude, hitherto we report the first known incidence of transient vision loss due to ACIOL coating by triamcinolone acetonide particles in the treatment of pseudophakic CME by IVTA. Although the risk of such a complication increases in the presence of vitrectomy, zonular weakness and lack of posterior capsular support, the anterior segment structures are not adversely damaged by TA coating which spontaneously resolves. Ophthalmologists should be aware of such a complication arising with the use of IVTA where patients cannot afford implantable steroids without worrying about the risk of serious effects of TA coating in ACIOL/scleral-fixated IOL pseudophakes.

## Notes

### Patient consent

Patient consent was obtained to publish these findings and images.

### Competing interests

The authors declare that they have no competing interests.

## References

[1] Chu CJ, Johnston RL, Buscombe C, Sallam AB, Mohamed Q, Yang YC, United Kingdom Pseudophakic Macular Edema Study Group (2016). Risk Factors and Incidence of Macular Edema after Cataract Surgery: A Database Study of 81984 Eyes. Ophthalmology.

[2] Perente I, Utine CA, Ozturker C, Cakir M, Kaya V, Eren H, Kapran Z, Yilmaz OF (2007). Evaluation of macular changes after uncomplicated phacoemulsification surgery by optical coherence tomography. Curr Eye Res.

[3] Kwong YY, Yuen HK, Lam RF, Lee VY, Rao SK, Lam DS (2007). Comparison of outcomes of primary scleral-fixated versus primary anterior chamber intraocular lens implantation in complicated cataract surgeries. Ophthalmology.

[4] Koutsandrea C, Moschos MM, Brouzas D, Loukianou E, Apostolopoulos M, Moschos M (2007). Intraocular triamcinolone acetonide for pseudophakic cystoid macular edema: optical coherence tomography and multifocal electroretinography study. Retina.

[5] Moshfeghi AA, Scott IU, Flynn HW, Puliafito CA (2004). Pseudohypopyon after intravitreal triamcinolone acetonide injection for cystoid macular edema. Am J Ophthalmol.

[6] Ruiz-Moreno JM, Montero JA, Artola A, Barile S (2005). Anterior chamber transit of triamcinolone after intravitreal injection. Arch Ophthalmol.

[7] Chen SD, Chen FK, Patel C (2006). Opaque coating of an intraocular lens and regression of iris neovascularization following injection of triamcinolone acetonide into the anterior chamber. Clin Exp Ophthalmol.

[8] Veritti D, Di Giulio A, Sarao V, Lanzetta P (2012). Drug safety evaluation of intravitreal triamcinolone acetonide. Expert Opin Drug Saf.

[9] Röck D, Bartz-Schmidt KU, Röck T (2019). Risk factors for and management of anterior chamber intravitreal dexamethasone implant migration. BMC Ophthalmol.

[10] Khurana RN, Appa SN, McCannel CA, Elman MJ, Wittenberg SE, Parks DJ, Ahmad S, Yeh S (2014). Dexamethasone implant anterior chamber migration: risk factors, complications, and management strategies. Ophthalmology.

[11] Qu Y, Liu XS, Liang AY, Xiao JY, Zhao C, Gao F, Zhang MF (2020). Subconjunctival injections of triamcinolone acetonide to treat uveitic macular edema. Int J Ophthalmol.

